# Microdebrider-Assisted Turbinoplasty Versus the Coblation Method of Turbinoplasty: A Comparative Study

**DOI:** 10.7759/cureus.82422

**Published:** 2025-04-17

**Authors:** Reshma Rajeev, Lathadevi H T, R N Karadi, Shashikumar T, Shivshankar Ajur

**Affiliations:** 1 Otorhinolaryngology- Head and Neck Surgery, Shri B M Patil Medical College, Hospital and Research Centre, Bijapur Lingayat District Education Association (BLDE) (Deemed to be University), Vijayapura, IND

**Keywords:** coblation, coblator assisted turbinoplasty, microdebrider, microdebrider assisted turbinoplasty, nasal obstruction, turbinate hypertrophy, turbinate reduction, turbinoplasty

## Abstract

Background

Inferior turbinate hypertrophy is a major cause of chronic nasal obstruction, significantly impairing nasal airflow and overall quality of life. Although medical management with antihistamines, decongestants, and corticosteroids is the first line of treatment, a subset of patients remains symptomatic and requires surgical intervention. Various surgical techniques, like mucosal sparing and non-mucosal sparing techniques, have become available recently. Microdebrider-assisted turbinoplasty (MAT) and coblation-assisted turbinoplasty (CAT) are two widely used mucosa-sparing techniques. However, limited comparative studies exist evaluating their efficacy, safety, and long-term outcomes. This study aims to compare the clinical outcomes of MAT and CAT in the surgical management of inferior turbinate hypertrophy.

Methods

This prospective comparative study included 60 patients diagnosed with symptomatic inferior turbinate hypertrophy unresponsive to medical therapy. Patients were assigned to undergo either MAT (n=30) or CAT (n=30) under general anesthesia using a lottery system. While the CAT group underwent controlled radiofrequency ablation with a coblator wand prior to outfracturing the turbinate, the MAT group underwent submucosal tissue removal and turbinate lateralization using a microdebrider. Postoperative outcomes were assessed using the Nasal Obstruction Symptom Evaluation (NOSE) score and objective airflow measurements at the seventh day, second, and third months following surgery. Additionally evaluated were intraoperative time, bleeding, complications, and postoperative healing.

Results

Both MAT and CAT showed significant improvements in NOSE scores, with mean scores improving from 72.4 ± 8.6 preoperatively to 18.7 ± 4.2 at three months in the MAT group and from 73.1 ± 7.9 to 19.3 ± 5.1 in the CAT group; however, there is no statistical difference between the two procedures in terms of symptomatic relief (p > 0.05). Peak nasal inspiratory flow (PNIF) improved by 62.3% in the MAT group and 58.7% in the CAT group at three months (p > 0.05). Intraoperative blood loss was slightly lower in the CAT group (21.5 ± 5.2 mL vs. 27.8 ± 6.4 mL in MAT, p < 0.05). Postoperative crusting and healing times were comparable between the two groups, with no significant difference in complication rates or recurrence of turbinate hypertrophy.

Conclusion

MAT and CAT are both effective and safe surgical options for managing inferior turbinate hypertrophy. While CAT offers a slight advantage in intraoperative hemostasis, both techniques provide comparable symptom relief, nasal airflow improvement, and mucosal preservation, making either a viable choice based on surgeon preference and patient-specific factors.

## Introduction

Inferior turbinate hypertrophy is one of the most common etiological causes of chronic nasal obstruction in patients seeking treatment at outpatient clinics. The inferior turbinates play a critical role in regulating nasal airflow and conditioning inspired air by filtering, warming, and humidifying it. However, when hypertrophied, they contribute significantly to nasal obstruction, leading to symptoms such as mouth breathing, dryness of the oral mucosa, nasal resonance changes, disturbed sleep, and reduced pulmonary function [1]. Turbinate hypertrophy is commonly associated with allergic rhinitis, vasomotor rhinitis, and chronic hypertrophic rhinitis. While medical management, including antihistamines, topical decongestants, and corticosteroids, is the first-line treatment, some patients remain refractory to these interventions and experience persistent nasal obstruction despite optimal medical therapy [2]​.

In cases where medical treatment fails to provide relief, surgical reduction of the inferior turbinate is necessary to improve nasal patency. A variety of surgical techniques are available to reduce the volume of both the mucosal and bony components of the inferior turbinate. These include cryosurgery, electrocautery, total or partial turbinectomy, turbinoplasty, and submucosal turbinectomy [3]. While these procedures generally yield satisfactory outcomes, they are also associated with postoperative complications such as bleeding, crust formation, pain, foul odor, synechiae formation, and, in some cases, atrophy of the inferior turbinates [4]. Additionally, procedures performed with traditional headlight illumination often fail to address hypertrophy at the posterior end of the turbinate, leading to persistent nasal obstruction in some cases. Although more aggressive surgical modalities, such as total or near-total turbinectomy, may offer more long-term relief, they carry a higher risk of complications, including excessive mucosal loss like atrophic rhinitis and empty nose syndrome [5].

To overcome these limitations, less destructive endoscopic procedures have been developed, utilizing advanced energy-based technologies such as lasers and radiofrequency to amplify precision and minimize trauma. Among these, radiofrequency ablation has gained widespread adoption due to its ability to effectively reduce submucosal turbinate volume while preserving the overlying mucosa, resulting in fewer postoperative complications and improved patient tolerance [6]. However, the ideal surgical technique for turbinate reduction remains unsettled, as each approach varies in its ability to balance the need for effective volume reduction while minimizing adverse effects such as bleeding, crusting, and prolonged recovery [7].

An optimal turbinate reduction procedure should address both the erectile submucosal tissue and the bony turbinate. While reducing the bony framework increases nasal airflow by enlarging the intranasal space, targeted submucosal tissue remodeling minimizes future mucosal engorgement. Additionally, preservation of the mucosal lining is essential for maintaining the physiological functions of the turbinates, including humidification, filtration, and temperature regulation [8]. Recent advancements have led to the increasing use of two mucosal-sparing surgical techniques: microdebrider-assisted turbinoplasty (MAT) and coblation-assisted turbinoplasty (CAT). These approaches have been developed to selectively remove hypertrophic tissue while preserving mucosal integrity, reducing complications, and improving long-term outcomes [9].

Turbinoplasty aims to remove the non-functional, obstructive portion of the turbinate while preserving the medial mucosa, which plays a primary role in conditioning inhaled air. Unlike more aggressive turbinectomy procedures, turbinoplasty provides a balanced approach by maintaining mucosal integrity while effectively reducing turbinate bulk [3]. The intraturbinal method, which is commonly used in MAT and CAT, primarily removes submucosal erectile tissue, leaving the bony inferior turbinate relatively intact. However, since the bony hypertrophy of the inferior turbinate also contributes to nasal obstruction, a modification known as the extraturbinal method has been developed. This approach combines the soft tissue resection of submucosal resection (SMR) with partial bony turbinate resection to enhance nasal airflow without compromising mucosal function​ [7,10].

MAT has gained popularity for its ability to provide precise tissue removal while minimizing trauma. The technique uses a powered microdebrider, which allows controlled resection of hypertrophic turbinate tissue under endoscopic guidance [11]. The microdebrider is commonly employed for extraturbinal procedures, where it selectively removes both hypertrophic soft tissue and portions of the inferior turbinate bone while sparing the overlying mucosa. This technique has demonstrated excellent postoperative outcomes in terms of nasal patency, with reduced complications compared to traditional turbinectomy [12]​.

CAT, on the other hand, utilizes bipolar radiofrequency energy to ablate submucosal hypertrophic tissue while preserving the overlying mucosa. Unlike conventional electrocautery, which operates at high temperatures and carries a greater risk of thermal damage, coblation technology operates at lower temperatures (40-70°C), reducing the risk of mucosal injury, crusting, and prolonged healing [13]. While coblation has been primarily used for intraturbinal procedures, its potential in extraturbinal applications remains relatively underexplored.

Despite the growing clinical adoption of MAT and CAT, a direct comparative analysis of their postoperative outcomes remains limited in the literature. While both techniques aim to achieve effective turbinate reduction with minimal complications, their relative efficacy, safety profiles, and long-term outcomes remain areas of ongoing investigation. Some studies suggest that MAT provides greater immediate airway relief due to mechanical tissue removal, while CAT may offer a more controlled, hemostatic approach with reduced intraoperative bleeding. However, the overall impact on long-term symptom relief, mucosal healing, and patient satisfaction has not been adequately compared [14].

Given the absence of a clear consensus on the superior technique, this study aims to compare the effectiveness and safety of MAT and CAT in the treatment of inferior turbinate hypertrophy resistant to medical therapy [15]. By evaluating key surgical outcomes, including postoperative nasal obstruction relief, mucosal healing, complication rates, and patient satisfaction, this study seeks to provide evidence-based insights to determine the optimal surgical approach for patients requiring turbinate reduction.

## Materials and methods

Study setting

This study is a hospital-based observational study conducted from April 2023 to January 2025 in the Department of Otorhinolaryngology at Shri B M Patil Medical College and Research Centre, Vijayapura, Karnataka. The Ethical Clearance Committee of Shri B. M. Patil Medical College Hospital and Research Centre, BLDE (Deemed to be University), Vijayapura, India, issued approval with IRB Number: BLDE(DU)/IEC/985/2022-23.

Inclusion criteria

A total of 60 patients were included in the study who were admitted for either MAT or CAT surgery using a lottery system after confirming the diagnosis and routine hematological and radiological evaluation (CT nose and paranasal sinuses). The anticipated mean ± SD of operation time in the coblation and microdebrider groups is 4.5 ± 0.14 and 4.9 ± 1.10. The required minimum sample size is 30 per group (i.e., a total sample size of 60, assuming equal group sizes) to achieve a power of 80% and a level of significance of 5% (two-sided) for detecting a true difference in means between two groups.

**Figure 1:**
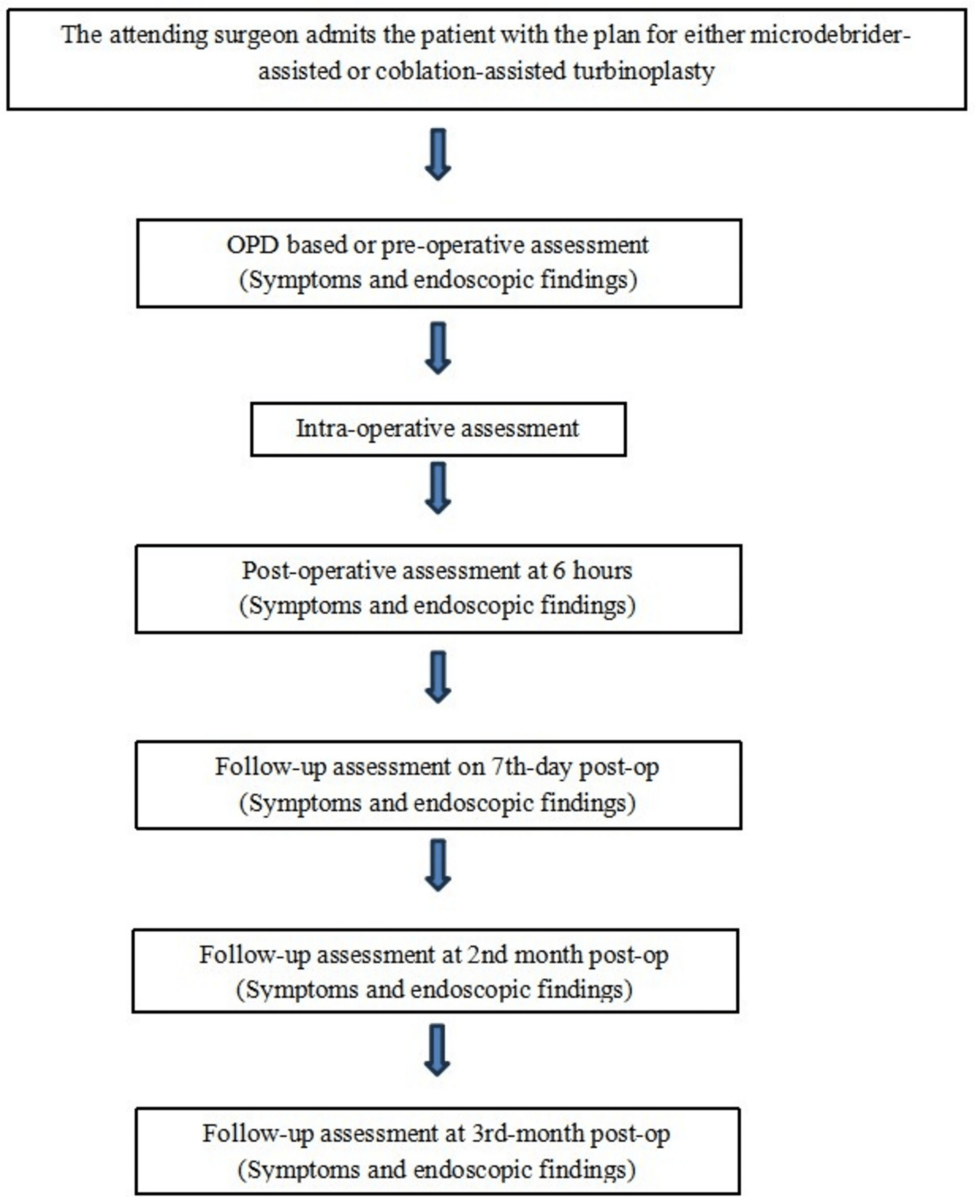
Study algorithm Turbinate size grading was done endoscopically according to Camacho et al. classification 2015 [15]. Symptom questionnaires were prepared according to the Nasal Obstruction Symptom Evaluation (NOSE) score. The image is created by author Reshma Rajeev.

The minimum duration of three months of nasal obstruction with clinical findings of inferior turbinate hypertrophy and patients with underlying conditions, such as seasonal allergies and mild to moderate deviated nasal septum (DNS), and patients who underwent septal surgeries in the past with persisting symptoms and turbinate hypertrophy were included. The study algorithm is depicted in Figure 1.

Exclusion criteria

Previous history of turbinate surgery, septal perforation or deformity, bleeding disorders, craniofacial malformations, or congenital malformations; patients with morbid obesity, maxillofacial trauma, tumors, granulomatous diseases of the nose, gross DNS, or chronic rhinosinusitis.

Statistical analysis

The collected data were initially entered into Microsoft Excel 2010 (Microsoft Corporation, Redmond, Washington, United States) for organization and cleaning. After ensuring accuracy and completeness, the dataset was imported into Jamovi Solid Version 2.3.28 (Computer Software, Retrieved from https://www.jamovi.org) for statistical analysis. The normality of continuous variables such as age, BMI, and vital parameters was assessed using Q-Q plots. Descriptive statistics were used to summarize the data, with continuous variables expressed as mean ± standard deviation (SD) or median with interquartile range (IQR), depending on their distribution. Categorical variables, including gender, weight groups, and visual function parameters, were presented as frequencies and percentages.

For inferential analysis, appropriate statistical tests were chosen based on the type and distribution of data. The normality of continuous variables such as intraoperative blood loss, surgical duration, and postoperative symptom scores was assessed using the Shapiro-Wilk test and visual inspection of Q-Q plots. Normally distributed variables were compared between groups using the independent t-test, with effect sizes calculated using Cohen’s d to quantify the magnitude of differences. Non-normally distributed continuous variables, including pain scores and nasal obstruction severity, were analyzed using the Mann-Whitney U test, with results expressed as median and interquartile range. Categorical variables, such as gender distribution, presence of synechiae, and postoperative bleeding, were assessed using the chi-square test or Fisher’s exact test when expected cell counts were less than five. The homogeneity of variances was tested using Levene’s test, ensuring the appropriate selection of parametric or nonparametric tests. Additionally, the chi-square test for trend was employed where applicable to assess the relationship between ordinal variables. All statistical tests were two-tailed, and a p-value of less than 0.05 was considered statistically significant.

Surgical procedure

All patients underwent surgery under general anesthesia. The patient was positioned in the reverse Trendelenburg position with 15-20 degrees of head-end elevation to facilitate venous drainage while ensuring adequate cerebral perfusion. The surgical site was painted and draped under sterile precautions.

Coblation-Assisted Turbinoplasty

CAT was performed by administration of local infiltration with 2% lignocaine with adrenaline and saline to both inferior turbinates for additional anesthesia and vasoconstriction. A coblator wand was introduced into the hypertrophied turbinate up to the third black marking and activated for 10 seconds to achieve controlled tissue ablation. The wand was then withdrawn progressively, with ablation performed at each black marking along the length of the turbinate for 10 seconds each. The turbinate was subsequently lateralized to further improve nasal airway patency. Postoperative nasal packing was not required, as intraoperative bleeding was minimal.

Microdebrider-Assisted Turbinoplasty

MAT was performed using the same preparation steps, infiltrating the local infiltration with 2% lignocaine with adrenaline and saline to both inferior turbinates. A microdebrider wand was introduced into the anterior end of the hypertrophied inferior turbinate and advanced posteriorly. Submucosal debridement was performed along the entire length of the turbinate while ensuring mucosal preservation. The lateralization of the inferior turbinate was done, and the same procedure was repeated on the opposite side.

Postoperatively, nasal packing was not required due to minimal blood loss. In cases where mild bleeding was anticipated, angel foam was applied to promote hemostasis. Both surgical techniques were performed with the objective of maximizing nasal airway improvement while minimizing mucosal trauma, ensuring optimal postoperative recovery and symptom relief.

## Results

**Figure 2:**
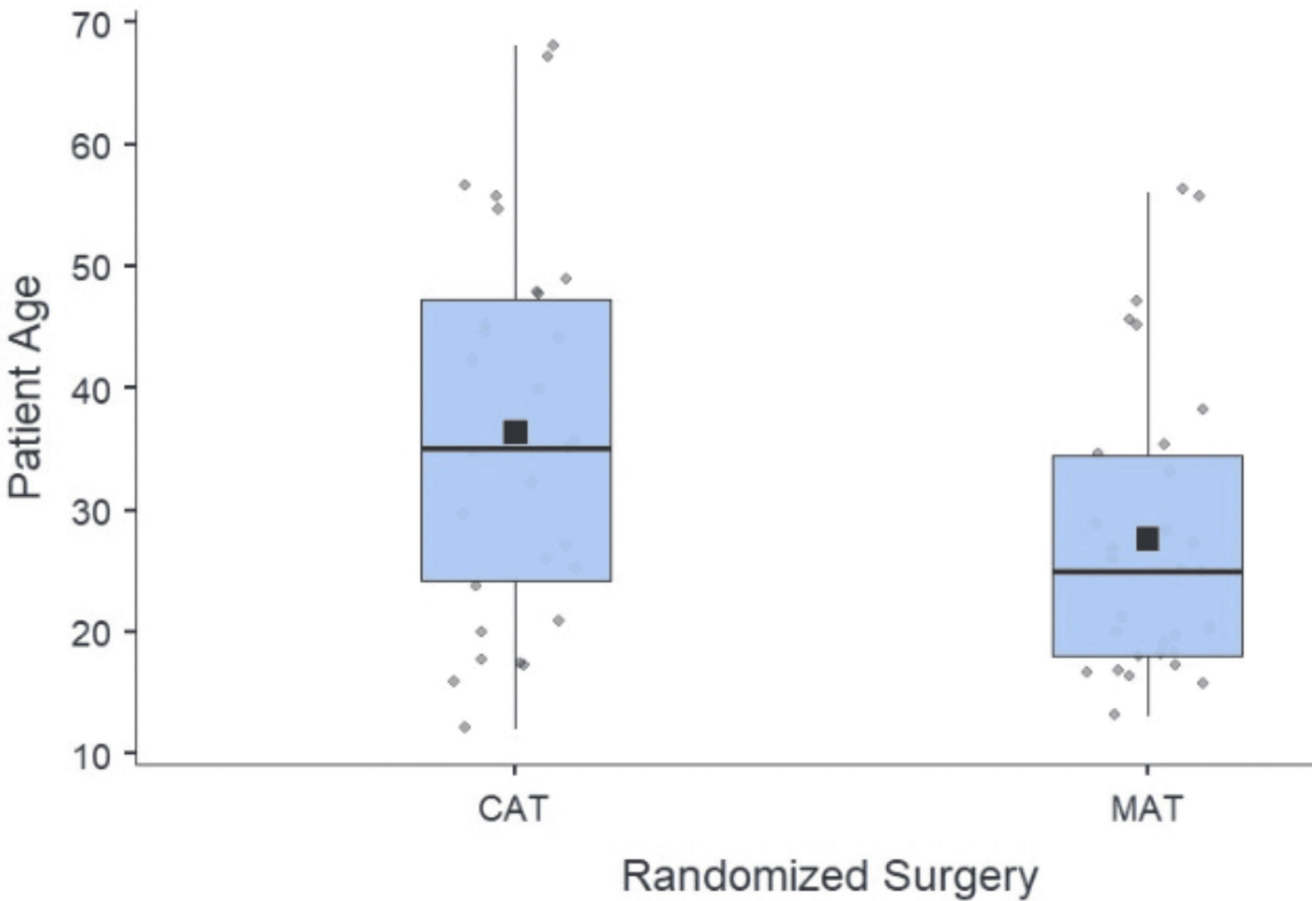
Age distribution of participants (N=60)

The age distribution of participants is summarized in Figure 2. The MAT group had a mean age of 27.6 ± 12 years, with a minimum age of 13 years and a maximum of 56 years. In contrast, the CAT group had a higher mean age of 36.3 ± 15.5 years, ranging from 12 to 68 years. Statistical analysis using an independent t-test revealed no significant difference between the two groups. This suggests that age was evenly distributed between the groups, supporting the validity of the randomization process. Hence, comparing outcomes between the MAT and CAT groups is unlikely to be influenced by age-related confounding.

**Figure 3:**
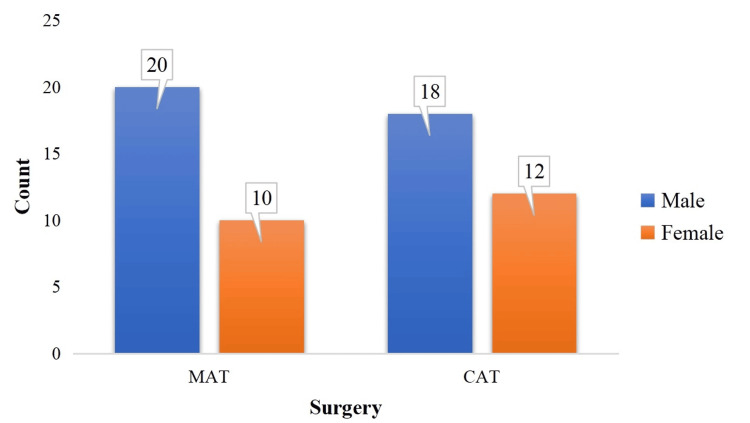
Distribution of gender across both surgery (N=60) MAT: microdebrider-assisted turbinoplasty; CAT: coblation-assisted turbinoplasty

The study included 60 participants, with a comparable distribution of males and females across both surgical groups. In the MAT group, 20 (66.7%) were male, and 10 (33.3%) were female. Similarly, in the CAT group, 18 (60%) were male, and 12 (40%) were female. A chi-square test for independence showed no statistically significant difference in gender distribution between the two groups, indicating that gender was evenly distributed. This supports the validity of randomization and ensures that gender-related factors are unlikely to influence the study outcomes (Figure 3).

The severity of pre-operative symptoms was assessed using a patient-reported rating scale from one to 10, with nasal obstruction being the most prominent symptom, having a mean score of 8.3 ± 1.2, ranging from six to 10. Headache was the second most reported symptom, with a mean score of 2.5 ± 2.5, ranging from zero to nine. Other symptoms, such as sneezing (0.5 ± 1, range: 0-4), rhinorrhea (0.35 ± 1, range: 0-3), and hyposmia (0.25 ± 0.7, range: 0-4) were reported less frequently and with lower severity. Snoring (1.6 ± 2, range: 0-7) and dry mouth upon waking or sleeping with the mouth open (1.5 ± 1.7, range: 0-7) were also observed in some patients, though with considerable variability in intensity.

The high severity of nasal obstruction highlights its significant impact on the study population, making it the primary complaint among participants. In contrast, other symptoms were present in a smaller proportion of patients and varied in intensity. Symptoms like snoring and dry mouth suggest associated airway compromise, though to a lesser extent than nasal obstruction. The variability in reported symptom scores indicates a heterogeneous symptom burden among participants. These baseline scores provide a crucial reference for assessing postoperative symptom improvement and the effectiveness of surgical intervention (Table 1).

**Table 1:** Pre-operative symptom scores (N=60)

Symptoms	Mean ± SD	Minimum	Maximum
Nasal obstruction	8.3 ± 1.2	6	10
Sneezing	0.5 ± 1	0	4
Rhinorrhea	0.35 ± 1	0	3
Hyposmia	0.25 ± 0.7	0	4
Headache	2.5 ± 2.5	0	9
Snoring	1.6 ± 2	0	7
Dry mouth upon waking/sleeping with the mouth open	1.5 ± 1.7	0	7

**Figure 4:**
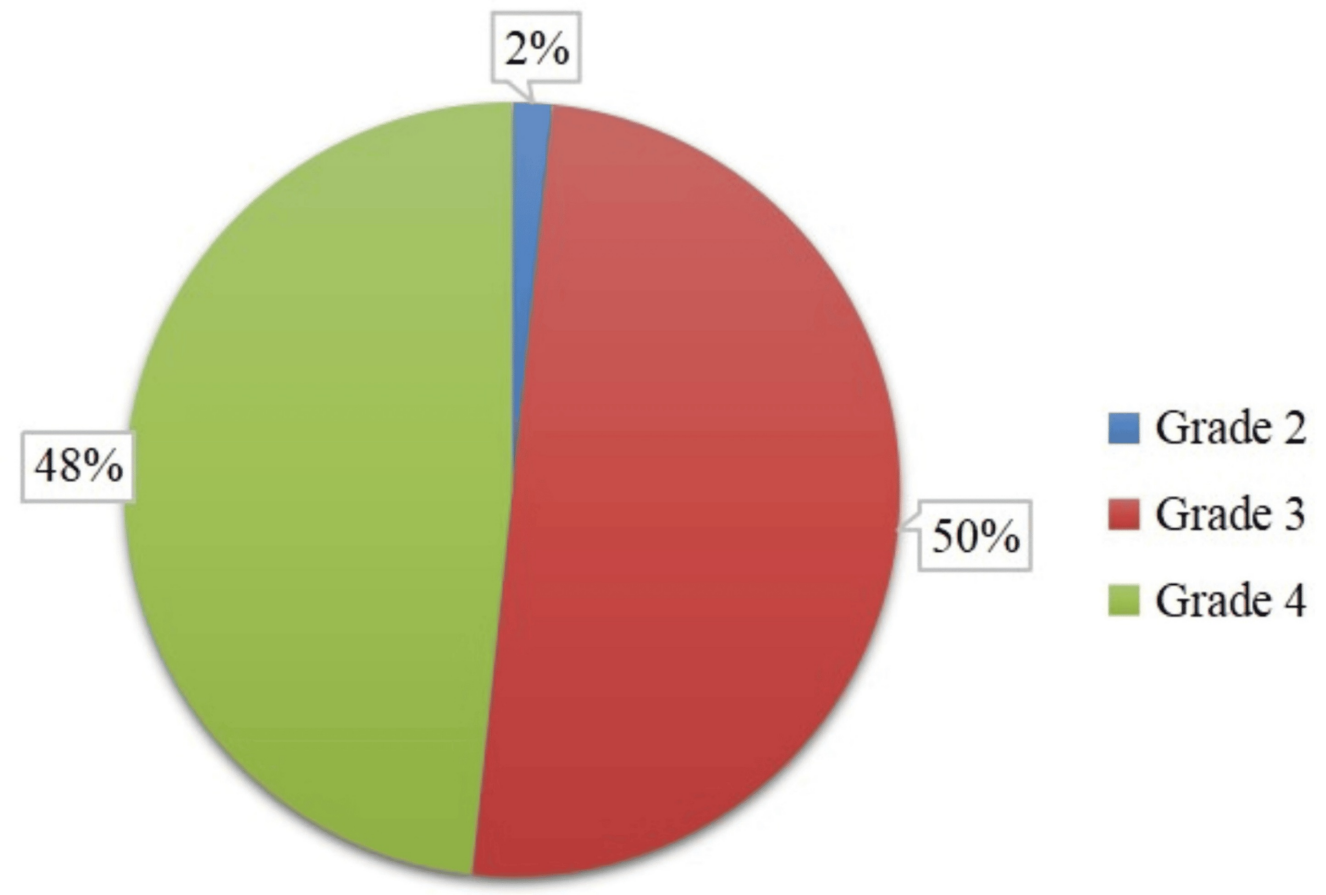
Pre-operative inferior turbinate size grading in nasal endoscopy (N=60)

Nasal endoscopy findings showed that most participants had moderate to severe inferior turbinate hypertrophy, with 30 (50.0%) classified as grade 3 and 29 (48.3%) as grade 4. Only one (1.7%) patient had grade 2 turbinate hypertrophy, indicating that most of the study population experienced significant nasal obstruction. The near-equal distribution of grade 3 and grade 4 turbinate hypertrophy suggests a substantial disease burden, emphasizing the necessity of surgical intervention for symptom relief. These baseline findings are a reference for assessing postoperative outcomes regarding turbinate size reduction and nasal airflow improvement (Figure 4).

**Figure 5:**
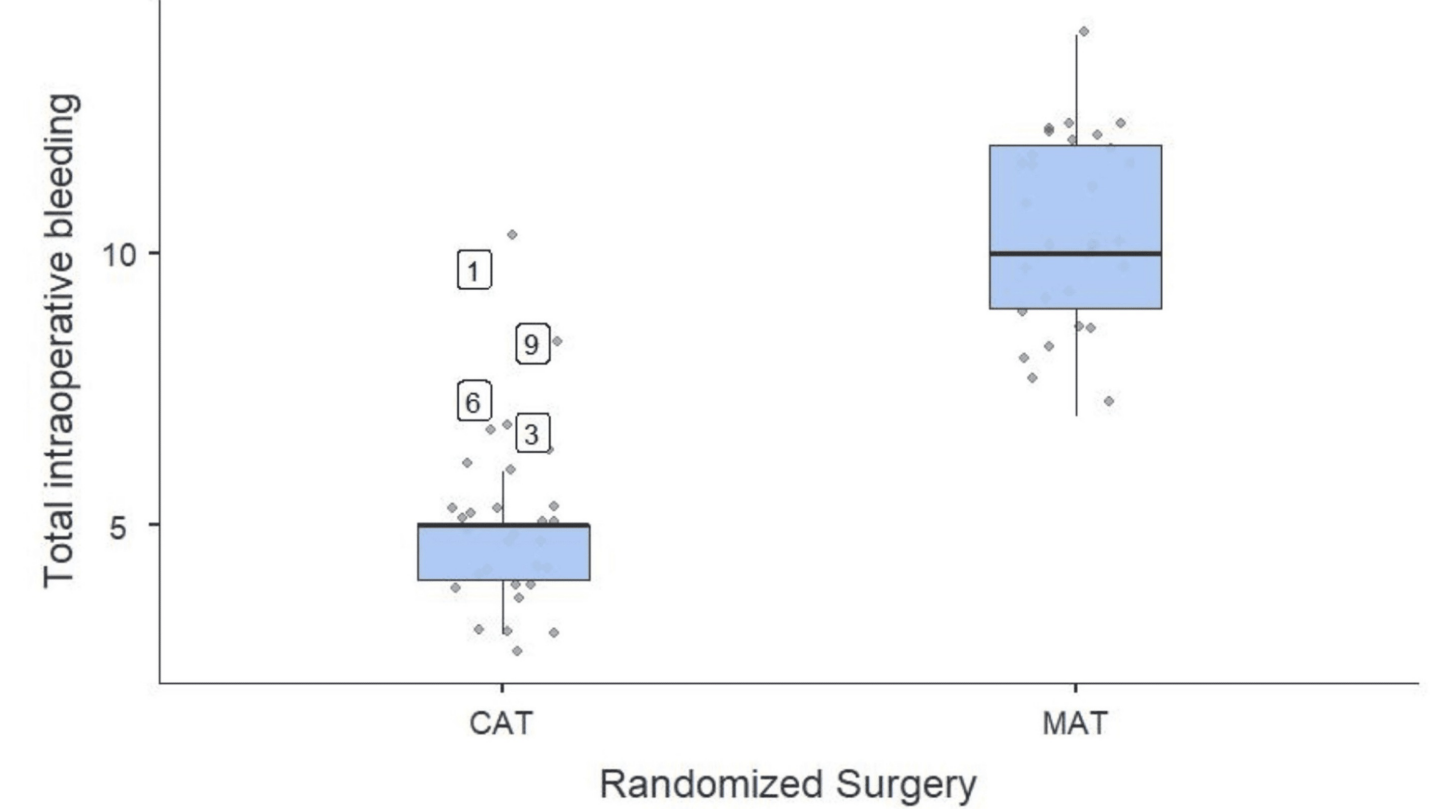
Comparison of total intraoperative blood loss (in ml) (N=60) MAT: microdebrider-assisted turbinoplasty; CAT: coblation-assisted turbinoplasty

The comparison of the two surgical techniques in terms of intraoperative blood loss revealed a significant difference. Patients who underwent MAT had a mean blood loss of 10.5 ± 1.7 ml. The CAT group, on the other hand, had significantly less bleeding, with an average of 5 ± 1.5 ml. According to statistical analysis, this difference had a significant effect size (Cohen’s d=3.76) and was highly significant (p < 0.05). The substantially lower blood loss in the CAT group suggests that the coblation method offers a superior hemostatic effect compared to the microdebrider technique. This reduction in intraoperative bleeding may contribute to better surgical visibility, reduced operative time, and potentially improved postoperative recovery (Figure 5).

**Figure 6:**
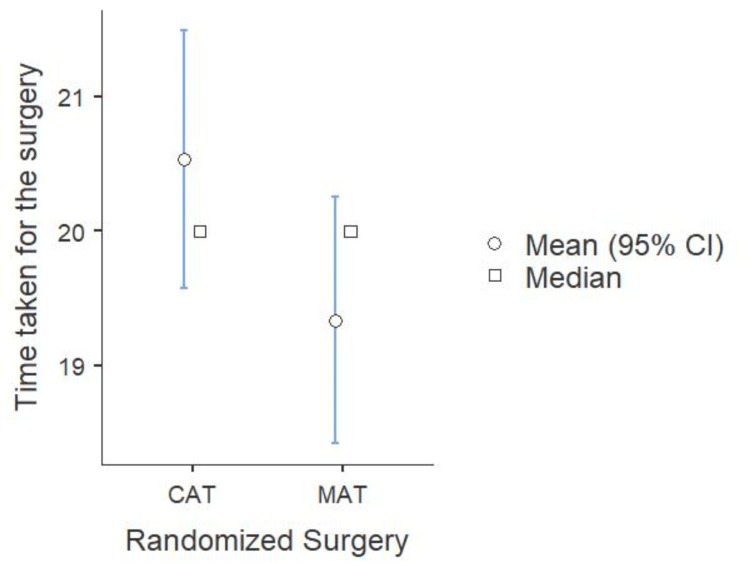
Comparison of total time taken across both surgeries (N=60) MAT: microdebrider-assisted turbinoplasty; CAT: coblation-assisted turbinoplasty

Figure 6 compares the two groups’ total surgery duration. The median surgical time for MAT was 20 minutes (IQR: 18-22), while for CAT, it was also 20 minutes (IQR: 20-22). The surgical times of the two procedures did not differ significantly, according to statistical analysis using the Mann-Whitney test (p=0.124). These results imply that both approaches take about the same amount of time to complete, suggesting that methodology selection has no bearing on the total length of the procedure.

**Figure 7:**
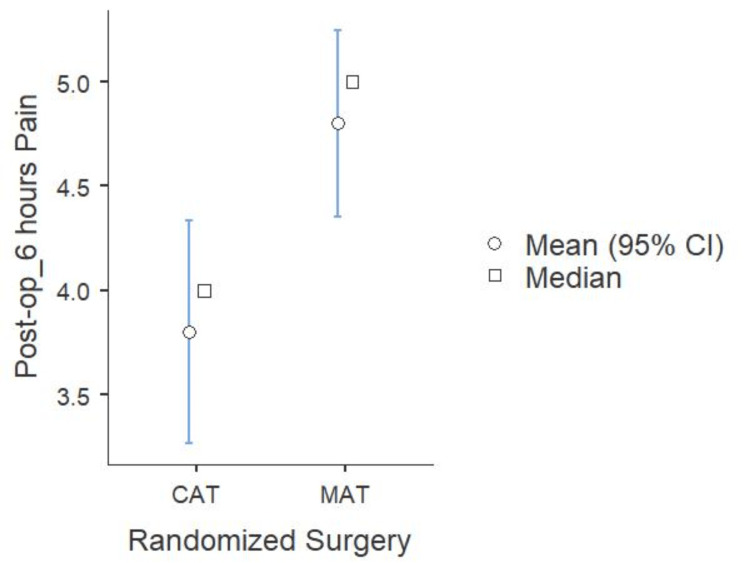
Comparison of six-hour postoperative pain score across both surgeries (N=60) MAT: microdebrider-assisted turbinoplasty; CAT: coblation-assisted turbinoplasty

Patients who underwent MAT reported higher postoperative pain scores at six hours than those who underwent CAT. The mean pain score in the MAT group was 5 ± 1.2, while in the CAT group, it was 4 ± 1.5. A moderate difference in pain levels was indicated by statistical analysis, which revealed a significant difference between the groups (p < 0.05) with an effect size of 0.727. These results imply that the coblation technique can enhance early recovery and postoperative pain management (Figure 7).

**Figure 8:**
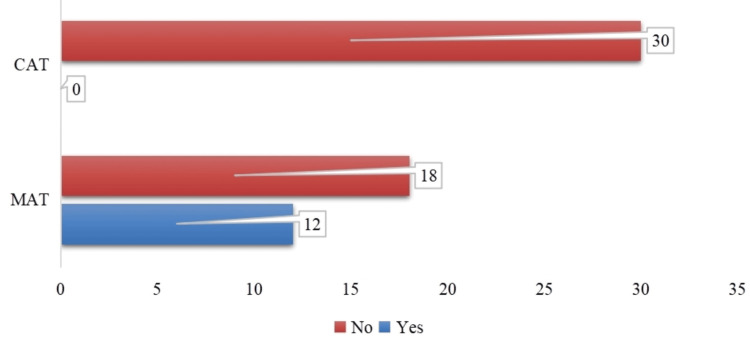
Comparison of six-hour postoperative bleeding across both surgeries (N=60) x-axis: number of patients who had six-hour postoperative blood loss (blue: blood loss present, red: no blood loss); y-axis: randomized surgery MAT: microdebrider-assisted turbinoplasty; CAT: coblation-assisted turbinoplasty

Twelve patients (40%) in the MAT group experienced postoperative bleeding, whereas the remaining 18 patients (60%) did not. This was a significant difference between the groups. On the other hand, all 30 patients (100%) in the CAT group did not experience any bleeding after the procedure. Statistical analysis established a significant difference between the two methods using Fisher’s exact test (p < 0.05). The CAT group’s total lack of postoperative bleeding highlights the coblation method’s superior hemostatic impact. It raises the possibility of a benefit in lowering the risk of bleeding-related postoperative sequelae (Figure 8).

Table 2 compares postoperative symptom scores on the seventh day between the two surgical techniques. Pain scores were significantly lower in the CAT group, with a median score of zero (IQR: 0-1), compared to two (IQR: 1-2) in the MAT group (p < 0.05), indicating a clear advantage of the coblation method in reducing postoperative discomfort. Nasal obstruction remained slightly higher in the MAT group, with a median score of four (IQR: 4-5), while in the CAT group, it was lower at three (IQR: 2-4), though the difference was not statistically significant (p=0.173). Both groups’ symptoms, including sneezing, rhinorrhea, hyposmia, snoring, and dry mouth upon waking, were absent, indicating good postoperative recovery. Headache scores were comparable between the two groups, with a median of 0.5 in both, though with a slightly broader range in the CAT group (IQR: 0-2) compared to the MAT group (IQR: 0-1), and the difference was not statistically significant (p=0.873). These findings suggest that both techniques lead to symptom resolution over time; the coblation method offers a notable advantage in reducing postoperative pain.

**Table 2:** Seventh-day postoperative symptom scores (N=60) MAT: microdebrider-assisted turbinoplasty; CAT: coblation-assisted turbinoplasty

Symptoms	Randomized Surgery	Median (IQR)	P-value
Pain	MAT	2 (1 - 2)	< 0.001
	CAT	0 (0 - 1)	
Nasal obstruction	MAT	4 (4 - 5)	0.173
	CAT	3 (2 – 4)	
Sneezing	MAT	0	-
	CAT	0	
Rhinorrhoea	MAT	0	-
	CAT	0	
Hyposmia	MAT	0	-
	CAT	0	
Headache	MAT	0.5 (0 - 1)	0.873
	CAT	0.5 (0 – 2)	
Snoring	MAT	0	-
	CAT	0	
Dry mouth upon waking/sleeping with the mouth open	MAT	0	-
	CAT	0	

**Figure 9:**
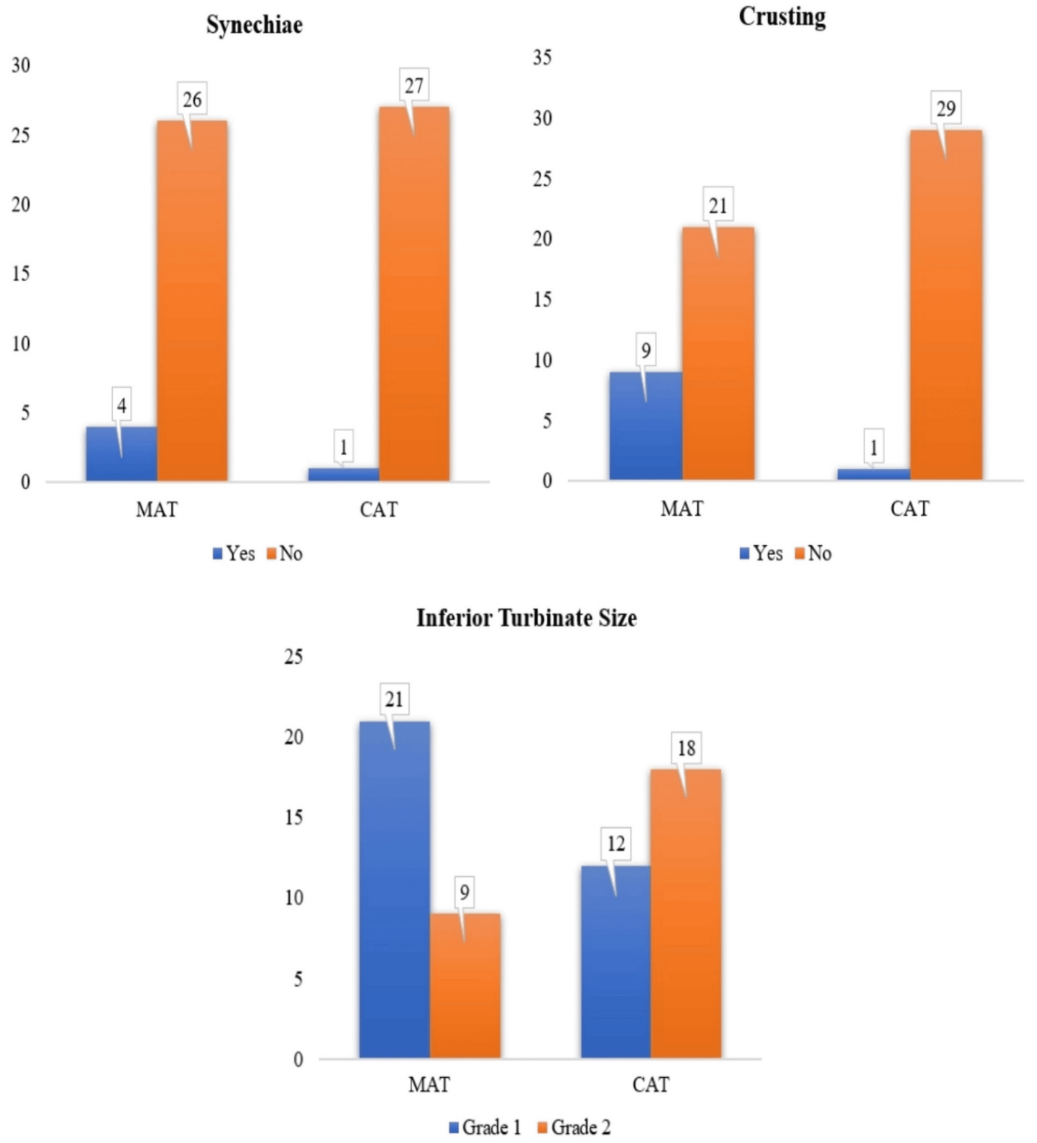
Comparison of seventh-day postoperative nasal endoscopy findings across both surgeries (N=60) x-axis: randomised surgery; y-axis: number of patients who had seventh postoperative day complications like synechiae and nasal crusting in each group, and reduction in the turbinate size MAT: microdebrider-assisted turbinoplasty; CAT: coblation-assisted turbinoplasty

On the seventh postoperative day, nasal endoscopy findings revealed differences in healing outcomes between the two surgical techniques. Synechiae formation was observed in a small number of patients, with four cases (13.3%) in the MAT group compared to only one case (3.3%) in the CAT group. However, the difference was not statistically significant (p=0.353). Crusting was more frequent in the MAT group, affecting nine patients (30%), while it was significantly lower in the CAT group, with only one patient (3.3%) experiencing this complication (p < 0.05). These findings suggest that the coblation method may provide a smoother postoperative recovery by reducing excessive crusting. Regarding reducing inferior turbinate size, more patients in the MAT group achieved grade 1 turbinate size (21 patients, 70%) compared to the CAT group (12 patients, 40%). Conversely, grade 2 turbinate size was more common in the CAT group, affecting 18 patients (60%) compared to nine (30%) in the MAT group. This difference was statistically significant (p < 0.05), indicating that the microdebrider technique was more effective in reducing turbinate size. While both methods showed favorable postoperative outcomes, the microdebrider-assisted technique resulted in better turbinate size reduction, whereas the coblation method minimized postoperative complications such as synechiae and crusting (Figure 9).

At the two-month postoperative follow-up, symptom resolution was observed across both surgical groups, with most patients reporting no residual complaints. Compared to the seventh postoperative day, pain had wholly subsided in both groups, with a median score of zero. Similarly, symptoms such as sneezing, rhinorrhea, hyposmia, headache, snoring, and dry mouth upon waking remained absent in all patients, indicating sustained postoperative recovery with minimal long-term complications. Nasal obstruction showed notable improvement in both groups, but remained significantly lower in the CAT group. At two months, the median nasal obstruction score in the MAT group was two (IQR: 1-2), while in the CAT group, it was significantly lower at 0.5 (IQR: 0-1) (p < 0.05). This reflects a more sustained reduction in nasal obstruction in patients who underwent coblation, in contrast to the MAT group, where some nasal blockage persisted. Compared to the seventh postoperative day, both techniques showed substantial improvement in symptoms over time. While the pain was minimal by day seven, the significant difference in nasal obstruction at two months suggests that coblation may provide superior long-term relief from nasal congestion. Overall, both procedures demonstrated effective symptom resolution, though CAT appeared to offer a more pronounced reduction in nasal obstruction by the end of the follow-up period (Table 3).

**Table 3:** Second month postoperative symptom scores (N=60) MAT: microdebrider-assisted turbinoplasty; CAT: coblation-assisted turbinoplasty

Symptoms	Randomized Surgery	Median (IQR)	P-value
Pain	MAT	0	-
	CAT	0	
Nasal obstruction	MAT	2 (1 – 2)	< 0.001
	CAT	0.5 (0 – 1)	
Sneezing	MAT	0	-
	CAT	0	
Rhinorrhoea	MAT	0	-
	CAT	0	
Hyposmia	MAT	0	-
	CAT	0	
Headache	MAT	0	-
	CAT	0	
Snoring	MAT	0	-
	CAT	0	
Dry mouth upon waking/sleeping with the mouth open	MAT	0	-
	CAT	0	

**Figure 10:**
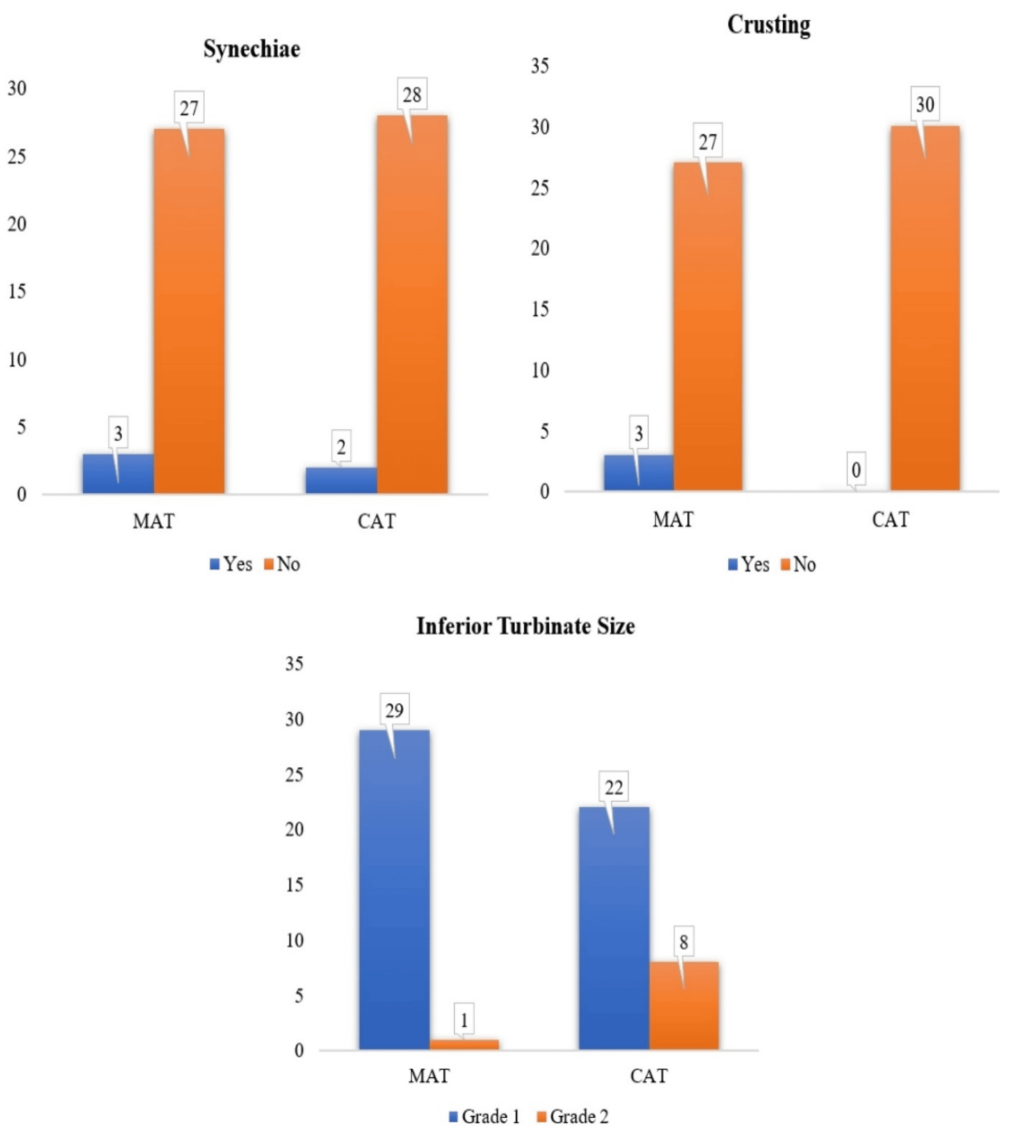
Comparison of second-month postoperative nasal endoscopy findings across both surgeries (N=60) x-axis: randomized surgery; y-axis: number of patients who had second-month postoperative complications like synechiae, nasal crusting, and reduction in turbinate size and grade of reduction. MAT: microdebrider-assisted turbinoplasty; CAT: coblation-assisted turbinoplasty

At the two-month postoperative follow-up, nasal endoscopy findings indicated further improvement in healing outcomes across both surgical techniques compared to the seventh postoperative day. Synechiae formation was minimal in both groups, with three cases (10%) in the MAT group and two cases (6.7%) in the CAT group, showing no significant difference (p=1.0). Crusting, which was more prevalent on day seven, had significantly reduced by two months. While three patients (10%) in the MAT group still exhibited minor crusting, there were no cases in the CAT group, though the difference was not statistically significant (p=0.237). The complete resolution of crusting in the CAT group suggests superior mucosal healing over time compared to the MAT group. Inferior turbinate size reduction remained significantly different between the two groups. Grade 1 turbinate size was achieved in a higher proportion of patients in the MAT group (29 patients, 96.7%) compared to the CAT group (22 patients, 73.3%), while grade 2 turbinate size was more common in the CAT group (eight patients, 26.7%) than in the MAT group (one patient, 3.3%) (p < 0.05). These findings indicate that while both techniques resulted in sustained improvement in turbinate size over time, the microdebrider technique remained superior in achieving more significant turbinate size reduction. Overall, by the two-month follow-up, both methods showed substantial resolution of synechiae and crusting, with coblation demonstrating better healing outcomes and microdebrider achieving more significant turbinate reduction (Figure 10).

**Figure 11:**
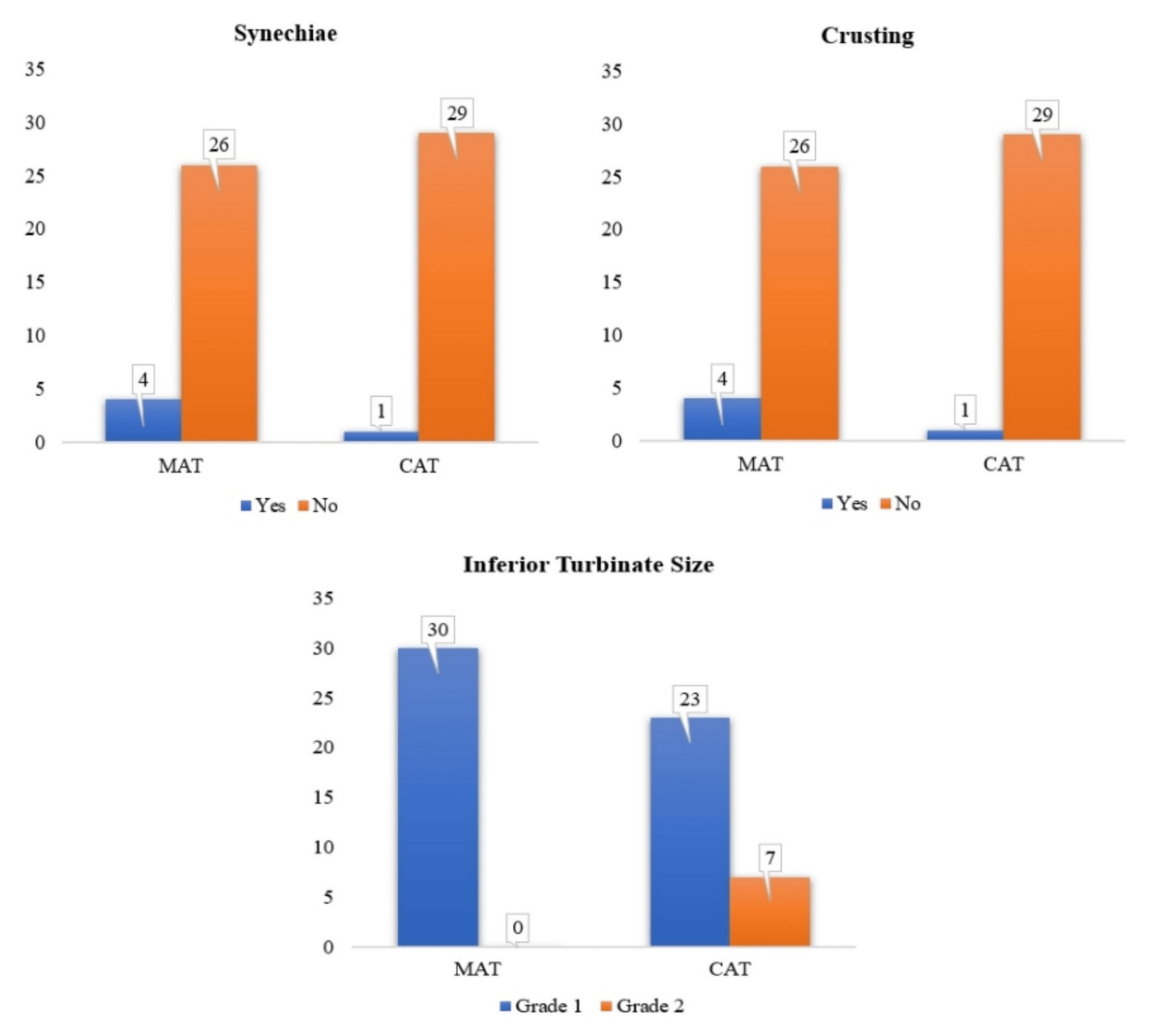
Comparison of third-month postoperative nasal endoscopy findings across both surgeries (N=60) x-axis: randomized surgery; y-axis: number of patients who had third-month postoperative complications like synechiae, nasal crusting, and postoperative turbinate size reduction and grade of reduction. MAT: microdebrider-assisted turbinoplasty; CAT: coblation-assisted turbinoplasty

By the third postoperative month, nasal endoscopy findings demonstrated continued improvement in healing outcomes across both surgical techniques. The absence of crusting, a standard postoperative issue, in both groups by the third month is a reassuring sign of complete mucosal recovery and normalization of nasal surfaces. Synechiae formation remained minimal, with four patients (13.3%) in the MAT group and only one patient (3.3%) in the CAT group, showing no significant difference between the two groups (p=0.353). The MAT group demonstrated remarkable success in achieving grade 1 turbinate size, with all patients (30 patients, 100%) reaching this level. In contrast, in the CAT group, 23 patients (76.7%) achieved grade 1, while the remaining seven (23.3%) had grade 2 turbinate size. This difference was not just significant but statistically significant (p < 0.05), confirming the superior effectiveness of the microdebrider technique in achieving more significant turbinate size reduction than CAT. These findings confirm the complete resolution of crusting and minimal synechiae formation with both techniques and highlight the impressive long-term turbinate reduction achieved by the microdebrider method. (Figure 11).

## Discussion

MAT and CAT are two widely used surgical techniques for managing inferior turbinate hypertrophy, a common cause of nasal obstruction. While both procedures aim to alleviate nasal airway resistance and improve patient symptoms, they differ in their underlying mechanisms, intraoperative characteristics, and postoperative outcomes [16]. MAT employs a high-speed rotating blade to mechanically reduce the hypertrophied turbinate tissue, whereas CAT uses radiofrequency energy to achieve controlled tissue ablation with concurrent hemostasis. Given the distinct approaches of these techniques, a comprehensive comparison is essential to determine their relative efficacy and safety. This study evaluates the clinical outcomes of MAT and CAT in terms of symptom resolution, turbinate size reduction, intraoperative efficiency, and postoperative complications, providing insights that may guide clinicians in selecting the most appropriate intervention for patients with chronic nasal obstruction.

The study included a total of 60 participants, evenly distributed between the MAT and CAT groups. The mean age of participants in the MAT group was 27.6 ± 12 years, while the CAT group had a higher mean age of 36.3 ± 15.5 years. Pre-operative symptom assessment revealed that nasal obstruction was the most prevalent complaint. Other symptoms, such as headache and snoring, were also reported, albeit with lower intensity. Nasal endoscopy findings demonstrated that the majority of patients presented with grade 3 or 4 turbinate hypertrophy, highlighting the substantial disease burden in the study population.

Intraoperative outcomes showed a significant difference in blood loss between the two techniques, highlighting the superior hemostatic effect of CAT, which may contribute to better surgical visualization and reduced perioperative morbidity. However, the total duration of surgery was similar for both techniques. Postoperatively, patients in the CAT group experienced significantly lower pain scores at six hours compared to those in the MAT group. Furthermore, while 40% of patients in the MAT group experienced postoperative bleeding, none of the patients in the CAT group reported this complication, reaffirming the superior hemostatic properties of coblation. On the seventh postoperative day, pain remained significantly lower in the CAT group compared to the MAT group. Nasal obstruction showed a slight improvement in the CAT group, although the difference was not statistically significant. Crusting was significantly more frequent in the MAT group compared to the CAT group, indicating that coblation promotes better early mucosal healing. While synechiae formation was slightly higher in the MAT group than in the CAT group.

By the second postoperative month, symptom resolution was observed across both groups, with pain completely subsiding. Nasal obstruction remained significantly lower in the CAT group compared to the MAT group, suggesting better long-term nasal airflow improvement with coblation. Endoscopic findings showed minimal crusting and synechiae formation in both groups, with no statistically significant differences. However, a greater proportion of patients in the CAT group had residual grade 2 turbinate size compared to the MAT group. By the third postoperative month, both groups showed near-complete symptom resolution. Synechiae formation remained slightly higher in the MAT group than in the CAT group, though the difference was not significant. Crusting had fully resolved in both groups. The difference in turbinate size reduction persisted, with all patients in the MAT group achieving grade 1 turbinate size, whereas 23.3% of patients in the CAT group still had residual grade 2 hypertrophy. These findings indicate that while both techniques effectively alleviate nasal obstruction, MAT may offer superior anatomical turbinate size reduction, whereas CAT provides better long-term airflow improvement.

By the third postoperative month, both groups showed near-complete symptom resolution. Synechiae formation remained slightly higher in the MAT group (13.3%) than in the CAT group (3.3%), though the difference was not significant (p=0.353). Crusting had fully resolved in both groups. The difference in turbinate size reduction persisted, with all patients in the MAT group achieving grade 1 turbinate size, whereas 23.3% of patients in the CAT group still had residual grade 2 hypertrophy (p=0.011). These findings indicate that while both techniques effectively alleviate nasal obstruction, MAT may offer superior anatomical turbinate size reduction, whereas CAT provides better long-term airflow improvement.

Hegazy et al. examined 70 patients over six months, focusing on both subjective (visual analogue scale scores) and objective (turbinate size grading) outcomes [17]. Our study, with 60 participants, incorporated intraoperative and early postoperative assessments with follow-ups at seven days, two months, and three months. Both studies demonstrated significant symptom relief in both groups, though the patterns of recovery varied. In Hegazy et al., CAT provided earlier pain relief, with significantly lower pain scores at two days postoperatively (p=0.0001), consistent with our findings at six hours and seven days (p=0.007 and p < 0.001, respectively). Similarly, both studies found CAT to be superior in reducing postoperative crusting and bleeding, reinforcing its hemostatic advantage. Turbinate size reduction remained a differentiating factor. Hegazy et al. found comparable reductions between the groups at six months, whereas our study highlighted a more pronounced reduction with MAT at three months (p=0.011). This suggests that while CAT ensures early symptom relief, MAT may offer a more sustained anatomical change. Complications were fewer with CAT in both studies. Hegazy et al. noted a lower incidence of adhesions (5% vs. 16.7% in MAT), aligning with our findings that CAT significantly minimized postoperative bleeding (p < 0.001). Unlike Hegazy et al., our study also showed that CAT had superior long-term nasal obstruction relief at two months (p < 0.001), further supporting its role in enhancing airflow dynamics.

Similarly, our study and the research by Singh et al. (2020) evaluated the efficacy and safety of MAT and CAT in treating inferior turbinate hypertrophy [18]. Both studies found significant improvements in nasal symptoms and turbinate size reduction postoperatively for both techniques. However, Singh et al. reported no significant differences between MAT and CAT in terms of symptom relief and turbinate size reduction at various postoperative intervals, while our study observed a more pronounced reduction in turbinate size with MAT at three months (p=0.011) and superior nasal obstruction relief with CAT at two months (p < 0.001). Additionally, Singh et al. found a longer operating time for CAT compared to MAT (p=0.001), whereas our study did not find a significant difference in surgical time between the two procedures. Both studies reported minimal postoperative complications, suggesting that both MAT and CAT are safe and effective for treating inferior turbinate hypertrophy.

Jadhav et al. (2022) assessed the effectiveness of MAT and CAT for inferior turbinate hypertrophy, demonstrating significant improvements in nasal obstruction, turbinate size reduction, and mucociliary transit time [19]. Both studies used the Nasal Obstruction Symptom Evaluation (NOSE) score for evaluation, with Jadhav et al. reporting greater improvement with coblation at all follow-ups, while our study found comparable outcomes initially but superior long-term relief with coblation. Turbinate size reduction was significant in both studies, though Jadhav et al. observed greater reduction with the microdebrider, a finding consistent with our results showing pronounced anatomical reduction at three months. Mucociliary transit time was better preserved with coblation in both studies, suggesting less thermal damage and better mucosal function. Postoperative complications were minimal, though early crusting and synechiae were slightly more frequent with the microdebrider, resolving with time. Jadhav et al. noted a small risk of mucosal atrophy with excessive coblation use, an observation not prominent in our study but still a consideration. Overall, both studies highlight that coblation offers faster symptom relief and better mucosal preservation, while the microdebrider provides more definitive turbinate size reduction, reinforcing the need for individualized surgical selection based on patient needs.

Bhagat et al. (2024) compared MAT and CAT for inferior turbinate hypertrophy, assessing outcomes such as nasal obstruction relief, turbinate size reduction, intraoperative bleeding, and postoperative recovery [20]. Both our study and Bhagat et al.'s study concluded that coblation and microdebrider techniques were more effective than submucous diathermy. Bhagat et al. reported that coblation had advantages in terms of reduced intraoperative bleeding and lower postoperative pain scores, findings consistent with our study, where coblation showed superior early postoperative comfort. Microdebrider, in contrast, achieved greater turbinate size reduction and better symptomatic improvement over time, a result echoed in our study, where microdebrider provided sustained anatomical reduction at later follow-ups. Postoperative crusting was initially higher with the microdebrider in both studies, but resolved over time. Bhagat et al. noted that the preference for submucous diathermy remains due to cost and ease of use, despite its lower efficacy, a point not directly addressed in our study but relevant for practical considerations. Both studies reaffirm that coblation offers better immediate symptom relief and reduced complications, while the microdebrider provides more definitive long-term anatomical improvement, supporting individualized procedural selection.

Our study and Kumar et al. (2016) found CAT and MAT to be equally effective in relieving nasal obstruction up to six months postoperatively, with no major difference in surgical time or early symptom resolution [21]. However, our study showed that CAT had a significant hemostatic advantage, reducing postoperative bleeding (p < 0.001), while Kumar et al. found no significant difference in bleeding rates. Additionally, we observed MAT achieving a more pronounced turbinate size reduction at three months (p=0.011), whereas CAT sustained better nasal obstruction relief at two months (p < 0.001). Both studies highlight the effectiveness of these techniques, with CAT excelling in early recovery and hemostasis, while MAT provides lasting turbinate volume reduction.

Limitations

Subjective responses from patients can bring bias to the study, a small sample size, surgeon variability, affordability and accessibility to surgical equipment, and no long-term follow-up in assessing patient satisfaction, mucosal healing, and recurrence rates to refine surgical decision-making further.

## Conclusions

In conclusion, both MAT and CAT significantly improved nasal obstruction and symptom relief. However, distinct advantages were observed with each technique. CAT demonstrated superior hemostatic control, lower postoperative pain, and reduced crusting, making it a preferred option for minimizing early postoperative complications and promoting faster recovery. In contrast, MAT achieved greater long-term turbinate size reduction, which may be beneficial in patients requiring more extensive tissue removal. The comparable symptom relief and sustained long-term benefits observed in both groups suggest that the choice between these methods can be tailored based on surgeon expertise, intraoperative conditions, and patient-specific factors.

## References

[1] Karamatzanis I, Kosmidou P, Ntarladima V, Catalli B, Kosmidou A, Filippou D, Georgalas C (2022). Inferior turbinate hypertrophy: a comparison of surgical techniques. Cureus.

[2] Yoo SH, Kim HY, Kim YA, Mo JH (2023). Radiofrequency ablation and microdebrider-assisted turbinoplasty: 5-year postoperative outcomes. J Rhinol.

[3] Ragab A, Elbanhawy O, Khashba A, AbdelAziz M, Elbanhawy O (2016). Microdebrider-assisted turbinoplasty against submucosal cauterization in inferior turbinate hypertrophy. Menoufia Medical Journal.

[4] Jourdy D (2014). Inferior turbinate reduction. Operative Techniques in Otolaryngology-Head and Neck Surgery.

[5] Hesham A, Badran H, Hussein A, Amin S, Salah M (2014). Intraturbinal versus extraturbinal microdebrider-assisted inferior turbinoplasty: Preliminary results. EJENTAS.

[6] Abdullah B, Singh S (2021). Surgical interventions for inferior turbinate hypertrophy: a comprehensive review of current techniques and technologies. Int J Environ Res Public Health.

[7] Zhang K, Pipaliya RM, Miglani A, Nguyen SA, Schlosser RJ (2023). Systematic review of surgical interventions for inferior turbinate hypertrophy. Am J Rhinol Allergy.

[8] Veit JA, Nordmann M, Dietz B (2017). Three different turbinoplasty techniques combined with septoplasty: prospective randomized trial. Laryngoscope.

[9] Lorenz KJ, Maier H (2013). Microdebrider-assisted inferior turbinoplasty. Minimally invasive technique for the treatment of nasal airway obstruction caused by enlarged turbinates (Article in German). HNO.

[10] Ali AR, Yousef GI, Hussein MA, Elawamry MI (2024). Comparative study between surgical partial inferior nasal turbinectomy and coblation-assisted inferior turbinate reduction in cases of inferior nasal turbinate hypertrophy. Egypt J Otolaryngol.

[11] Kanesan N, Norhayati MN, Hamid SS, Abdullah B (2022). Microdebrider-assisted inferior turbinoplasty versus other surgical techniques. Acta Otorhinolaryngol Ital.

[12] Lee KC, Cho JM, Kim SK, Lim KR, Lee SY, Park SS (2017). The efficacy of coblator in turbinoplasty. Arch Craniofac Surg.

[13] Al-Qudah M, Al-Mardeeni D (2013). How I do it? Microdebrider assisted inferior turbinoplasty. Pan Arab J Rhinol.

[14] Neskey D, Eloy JA, Casiano RR (2009). Nasal, septal, and turbinate anatomy and embryology. Otolaryngol Clin North Am.

[15] Camacho M, Zaghi S, Certal V (2015). Inferior turbinate classification system, grades 1 to 4: development and validation study. Laryngoscope.

[16] Berger G, Balum-Azim M, Ophir D (2003). The normal inferior turbinate: histomorphometric analysis and clinical implications. Laryngoscope.

[17] Hegazy HM, ElBadawey MR, Behery A (2014). Inferior turbinate reduction; coblation versus microdebrider - a prospective, randomised study. Rhinology.

[18] Singh S, Ramli RR, Wan Mohammad Z, Abdullah B (2020). Coblation versus microdebrider-assisted turbinoplasty for endoscopic inferior turbinates reduction. Auris Nasus Larynx.

[19] Jadhav SG, Mane BS, Naikwadi KB, Gavali RM (2022). A comparative study between cobblator and microdebrider assisted inferior turbinate reduction surgery. Int J Otorhinolaryngol Head Neck Surg.

[20] Bhagat PR, Bathla M, Doshi H, Solanki K, Gajjar R (2024). A study of comparison of outcomes of submucous diathermy, coblation and micro-debrider assisted inferior turbinoplasty in patients having inferior turbinate hypertrophy. Indian J Otolaryngol Head Neck Surg.

[21] Kumar A, Goyal A, Singh R (2016). Coblation vs microdebrider-assisted inferior turbinoplasty. AIJOC.

